# Protective effect of allicin on high glucose/hypoxia-induced aortic endothelial cells via reduction of oxidative stress

**DOI:** 10.3892/etm.2015.2708

**Published:** 2015-08-24

**Authors:** SHU-LI WANG, DE-SHAN LIU, ER-SHUN LIANG, YAN-HUA GAO, YING CUI, YU-ZHAO LIU, WEI GAO

**Affiliations:** 1 Department of Traditional Chinese Medicine, Qilu Hospital of Shandong University, Jinan, Shandong 250012, P.R. China; 2 Department of Geriatrics, Linyi People's Hospital, Linyi, Shandong 276003, P.R. China; 3 Shandong University School of Medicine, Qilu Hospital of Shandong University, Jinan, Shandong 250012, P.R. China; 4 Key Laboratory of Cardiovascular Remodeling and Function Research, Qilu Hospital of Shandong University, Jinan, Shandong 250012, P.R. China

**Keywords:** oxidative stress, allicin, NADPH oxidase 4, hypoxia-inducible factor-1α, 8-hydroxydeoxyguanosine

## Abstract

This study was designed to explore the protective effect of allicin on aortic endothelial cell injury induced by high glucose/hypoxia and to investigate the corresponding mechanisms. The primary-cultured murine aortic endothelial cells were subcultured. The third passage of cells was adopted and randomly divided into five groups: The normal group (NG), the mannitol group (MG), the high-glucose/hypoxia group (HG), the allicin group (AG) and the protein kinase C (PKC) inhibitor group (GG). The general morphology was observed under an inverted phase-contrast microscope and cell viability was assessed using the MTT assay. Intracellular reactive oxygen species (ROS) levels in the endothelial cells were quantified using dihydroethidium staining. The levels of 8-hydroxydeoxyguanosine (8-OHdG), nuclear factor-κB (NF-κB), NADPH oxidase 4 (Nox4) and hypoxia-inducible factor-1α (HIF-1α) and the activity of PKC were measured using ELISA. A quantitative polymerase chain reaction (qPCR) was adopted to evaluate the mRNA expression of Nox4, HIF-1α and NF-κB. The altered cell morphology observed in HG was notably ameliorated in the AG and GG. The protein levels of 8-OHdG, NF-κB, Nox4, HIF-1α and PKC in the HG were higher than those in the other groups. Furthermore, the cell viability in the AG was significantly increased and the protein levels of 8-OHdG, NF-κB, Nox4, HIF-1α and PKC were significantly decreased compared with those in the HG. The ROS production was found to be increased in the HG cells, while there was a significant decrease in the AG cells. These data indicate that allicin exerts a protective effect against high glucose/hypoxia-induced injury in aortic endothelial cells through its antioxidative action, which may involve the inhibition of the PKC pathway and regulation of HIF-1α.

## Introduction

Diabetes mellitus is a serious social health problem, with diabetes-related vascular complications representing a major public health burden. According to statistics, >40% of patients hospitalized with acute myocardial infarction have clinical diabetes, and another 35% exhibit impaired glucose tolerance (1). Excessive production of reactive oxygen species (ROS) and the subsequent increase in oxidative stress under high-glucose conditions play a critical role in this pathology (2). The overproduction of ROS, including superoxide anion radicals, hydrogen peroxide and hydroxyl radicals, can prominently damage nucleic acids, lipids and proteins and result in cellular necrosis, tissue structural damage and functional disorder (3). In the setting of hypoxia, hypoxia-inducible factor-1α (HIF-1α) increases in response to ischemia. HIF-1α is a key regulator of oxygen homeostasis, which restores blood flow to ischemic regions (4); however, sustained and prolonged activation of the HIF-1α pathway induces cell death due to the subsequent activation of p53 and other associated genes (5).

Among the currently available medications for diabetes, natural products have drawn increasing attention. As a traditional Chinese herbal medicine, garlic has been used to treat a range of diseases for thousands of years. Allicin is one of the main active components in garlic and exerts several therapeutic effects, such as promoting insulin sensitivity (6,7), decreasing blood glucose levels (8,9), regulating lipid metabolism (10,11), reducing homocysteine levels (12), attenuating superoxide production (13) and limiting inflammation and fibrogenesis. Given the role of oxidative stress within the etiology and pathogenesis of diabetic complications, the present study utilized an *in vitro* model of diabetes-associated oxidative stress using aortic endothelial cells cultured under high-glucose/hypoxic conditions to clarify the possible mechanism of allicin on diabetic macrovascular complications.

## Materials and methods

### 

#### Materials

The experiment was carried out in the Institute of Basic Medical Sciences, Qilu Hospital of Shandong University (Jinan, China) between February and September, 2014. The murine aortic endothelial cells were obtained from CHI Scientific, Inc. (Jiangyin, China). Xanthine (X), xanthine oxidase (XO), MTT, trypsin, and GF109203x [an inhibitor of protein kinase C (PKC)] were purchased from Sigma-Aldrich (St. Louis, MO, USA). Allicin was provided by Xuzhou Laien Pharmaceutical Co. Ltd. (Xuzhou, China). Endothelial cell medium (ECM) was provided by Sciencell Research Laboratories (Carlsbad, CA, USA) and TRIzol® was obtained from Gibco-BRL (Grand Island, NY, USA). Culture flasks and 6-, 12-, 24- and 96-well culture plates were purchased from Sigma-Aldrich. 8-Hydroxydeoxyguanosine (8-OHdG), nuclear factor-κB (NF-κB), NADPH oxidase 4 (Nox4), HIF-1α and PKC test kits were obtained from Nanjing Jiancheng Bioengineering Institute (Nanjing, China). Taq DNA polymerase and oligo (dt) were provided by Fermentas (Thermo Fisher Scientific, Pittsburgh, PA, USA). The primers were synthesized by Sangon Biotech, Co., Ltd. (Shanghai, China), and all other reagents were analytically pure.

#### Cell cultures

Murine aortic endothelial cells of the third passage were used in this study. Cells were grown in ECM at 37°C with 5% CO_2_ and 95% air. The medium was changed every 2 days, and the cells were checked every day using an inverted phase-contrast microscope (CKX41; Olympus Corp., Tokyo, Japan). When the cells had predominantly mixed together and reached ~80% confluence, the medium was abandoned and the cells were gently washed twice with phosphate-buffered saline (PBS), prior to detachment from the culture flask, with the aid of 0.2% (w/v) trypsin for 2–3 min. A single cell suspension was generated, and the cells were plated in 6-, 12-, 24- or 96-well plates.

#### Grouping and treatments

Allicin injection, X/XO and GF109203x were prepared in serum-free medium at 10 µg/ml, 1 mmol/l/20 U/l and 10 µmol/l, respectively. The murine aortic endothelial cells in the exponential growth phase were randomly divided into five groups: The normal group (NG), the mannitol group (MG), the high-glucose/hypoxia group (HG), the allicin group (AG) and the PKC inhibitor (GF109203x) group (GG). The cells of the NG and MG were respectively incubated with serum-free ECM and mannitol (25 mmol/l) for 24 h. The cells of the HG, AG and GG were respectively incubated with X/XO (1 mmol/l/20 U/l), allicin (10 µg/ml) plus X/XO (1 mmol/l/20 U/l), and GF109203x (10 µmol/l) plus X/XO (1 mmol/l/20 U/l) for 40 min, prior to the supernatant solution being abandoned and the cells being treated with glucose (25 mmol/l), allicin (10 µg/ml) plus glucose (25 mmol/l), and GF109203x (10 µmol/l) plus glucose (25 mmol/l), respectively, for 24 h. Following each of the above treatment regimens, the supernatant and culture cells were collected. The collected cells were resuspended in PBS. In order to measure the components within cells, the freeze-thawing method was adopted to break up the cells.

#### Cell viability assay

Following the different treatments, the morphological changes were observed under a phase-contrast microscope (Olympus Corp.). In addition, MTT assays were performed to assess the cell viability. First, cell viability was assessed using MTT when the cells were cultured in different allicin concentrations of 50, 20, 10, 5 and 2.5 µg/ml (all diluted in serum-free ECM). In brief, the cells of the six groups (the normal control group and the five groups of different allicin concentrations) were seeded into 96-well plates at a density of 1×10^4^ cells per well. Upon reaching ~80% confluence, the cells were incubated with different concentrations of allicin for 24 h. The supernatant was then abandoned, and 200 µl serum-free medium, as well as 20 µl MTT dye solution (5 mg/ml), was added to each well. Following the incubation of the samples at 37°C for 4 h, the MTT/medium solution was removed and 150 µl dimethylsulfoxide was added to dissolve the formazan product in each well on a concentrating table for 15 min. The optical density of each well was measured using an ELISA microplate reader (Bio-Rad Laboratories, Inc., Hercules, CA, USA) at a wavelength of 290 nm. In addition, the cell viability in the NG, MG, HG, AG and GG was assessed using MTT following treatment with the different interventions for 24 h.

#### Dihydroethidium (DHE) staining to measure ROS

DHE, which is used as a relatively specific measurement for the superoxide anion, is an oxidative fluorescent dye that undergoes a two-electron oxidation to form the DNA-binding fluorophore ethidium bromide. The DHE (Vigorous Biotechnology Beijing Co., Ltd., Beijing, China) staining for superoxide was carried out as previously described (14). Briefly, the cells were treated with the aforementioned interventions for 24 h. Following the removal of the supernatant solution, the cells were cultured in DHE (10 µmol/l) diluted with PBS in a light-protected, humidified chamber at 37°C for 30 min. Once the cells had been washed twice with PBS to remove the uncombined fluorescence probe, fluorescent images were obtained using a fluorescence microscope. The mean fluorescence intensity was measured using Image-Pro Plus 6.0 software (Media Cybernetics, Inc., Rockville, MD, USA) for quantification. The generation of superoxide was demonstrated by red fluorescent labeling. Non-stained cells were used as a background control. The average of three DHE-stained images was taken as the value for each group.

#### ELISA

The levels of 8-OHdG, NF-κB, Nox4, HIF-1α and PKC in the cells were determined by ELISA, according to instructions of each assay kit. Aortic endothelial cells were treated as previously described for 24 h. Following the removal of the supernatant solution, the cells were washed twice with ice-cold PBS, scraped from the plate with trypsin and centrifuged at 1,301 rpm for 5 min at 4°C. The cells were resuspended in PBS. The freeze-thawing method was performed to break up the cells.

#### Reverse transcription-quantitative polymerase chain reaction (RT-qPCR)

RT-qPCR was performed in accordance with the method described in our previous study (15). Briefly, total RNA was extracted using TRIzol reagent, following the manufacturer's instructions (Gibco-BRL). cDNA was synthesized using a commercial reverse transcription kit (Fermentas; Thermo Fisher Scientific, Waltham, MA, USA). The sequences of the primers and cycle conditions were as follows: i) HIF-1α sense, 5′-TCA AGT CAG CAA CGT GGA AG-3′ and antisense, 5′-TAT CGA GGC TGT GTC GAC TG-3′ (ampliﬁcation product, 198 bp; 94°C for 30 sec, 59°C for 30 sec and 72°C for 1 min, for 35 cycles); ii) Nox4 sense, 5′-TAG CTG CCC ACT TGG TGA ACG-3′ and antisense, 5′-TGT AAC CAT GAG GAA CAA TAC CACC-3′ (ampliﬁcation product, 170 bp; 94°C for 30 sec, 59°C for 30 sec and 72°C for 1 min, for 30 cycles); iii) NF-κB sense, 5′-GTA TTG CTGTGC CTA CCC GAA AC-3′ and antisense, 5′-GTT TGA GAT CTG CCC TGA TGG TAA-3′ (ampliﬁcation product, 134 bp; 94°C for 30 sec, 55°C for 30 sec and 72°C for 1 min, for 30 cycles); iv) β-actin sense, 5′-TGG CAC CCA GCA CAA TGAA-3′ and antisense, 5′-CTA AGT CAT AGT CCG CCT AGA AGCA-3′ (ampliﬁcation product, 188 bp; 94°C for 30 sec, 59°C for 30 sec and 72°C for 1 min, for 30 cycles). The mean value of the replicates for each sample was calculated and expressed as the cycle threshold (Ct). The gene expression was then calculated as the difference (ΔCt) between the Ct value of the target gene and the Ct value of β-actin.

#### Statistical analysis

Data are presented as the mean ± standard deviation. Analysis of variance was used to compare the mean values of more than two groups. Differences were considered significant at P<0.05. All statistical calculations were performed using SPSS 13.0 software (SPSS, Inc., Chicago, IL, USA).

## Results

### 

#### General morphological observation under the inverted phase-contrast microscope

**Figure 1.**
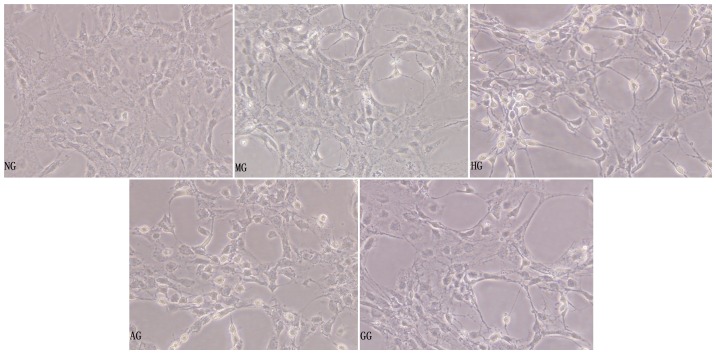
General morphological observation of aortic endothelial cells under the inverted phase-contrast microscope. Images are representative of each group (magnification, x200). NG, normal group; MG, mannitol group; HG, high glucose/hypoxia group; AG, allicin group; GG, PKC inhibitor (GF109203x) group.

Under high-glucose/hypoxic conditions for 24 h, the aortic endothelial cells became shrunken, the intercellular connection was lessened, some of the cells became exfoliated and a few of the cells were found to be floating in the supernate (Fig. 1, HG). The morphology of the MG cells was not obviously changed compared with that of the NG cells (Fig. 1, MG). Compared with the HG cells, the cells in the AG had more complete cell bodies with visible processes, indicating that allicin played a protective role in the injured cells (Fig. 1, AG).

#### Viability of aortic endothelial cells

**Figure 2.**
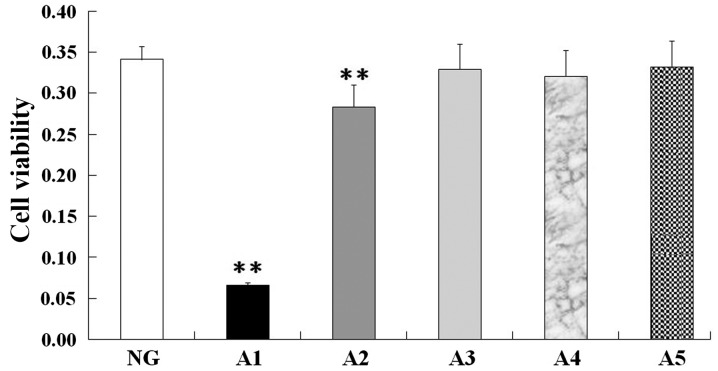
Effect of different concentrations of allicin on aortic endothelial cell viability. Data are expressed as the mean ± standard deviation (n=10 per group). **P<0.01 vs. NG. NG, normal group; A1, allicin at 50 µg/ml; A2, allicin at 20 µg/ml; A3, allicin at 10 µg/ml; A4, allicin at 5 µg/ml; A5, allicin at 2.5 µg/ml.

**Figure 3.**
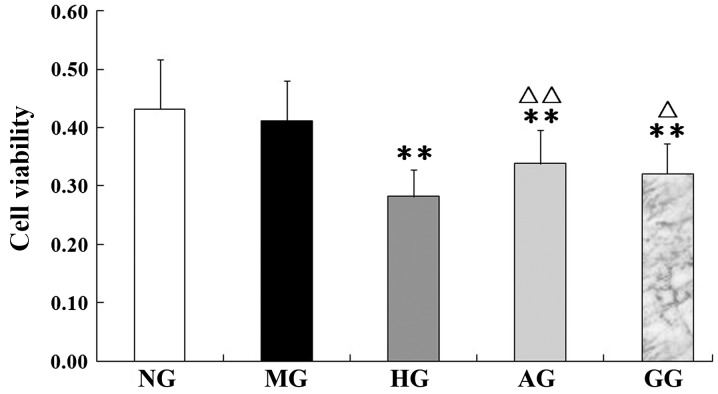
Cell viability of aortic endothelium treated with different interventions. Data are expressed as the mean ± standard deviation (n=14 per group). **P<0.01 vs. NG; ^∆^P<0.05 and ^∆∆^P<0.01 vs. HG. NG, normal group; MG, mannitol group; HG, high glucose/hypoxia group; AG, allicin group; GG, PKC inhibitor (GF109203x) group.

MTT assay revealed no significant differences in cell viability between the normal control group and the 10, 5 and 2.5 µg/ml allicin groups (Fig. 2, A3, A4 and A5); however, the cell viability of the 50 and 20 µg/ml allicin groups was significantly decreased compared with that of the normal control group (P<0.01) (Fig. 2, A1 and A2). Based on these findings, 10 µg/ml allicin was selected as the final treatment concentration. In addition, as shown in Fig. 3, an evident decrease in viability was observed in the cells under high-glucose/hypoxic conditions compared with the cells of the NG (P<0.01); however, in experimental conditions with allicin or GF109203x the cell viability was significantly increased compared with that of the HG (P<0.01 or P<0.05). Allicin and GF109203x exhibited similar effects on cell viability.

#### Effect on ROS

**Figure 4.**
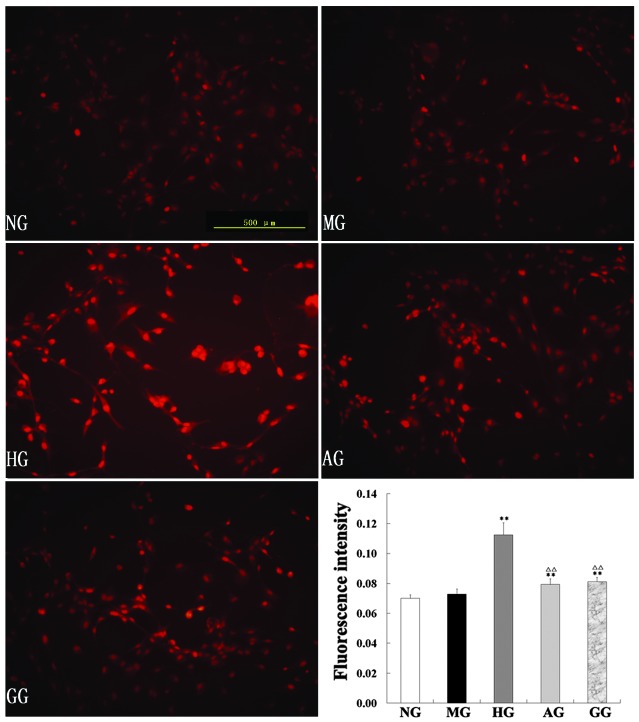
Reactive oxygen species levels of aortic endothelial cells assessed by dihydroethidium staining under a fluorescence microscope. Image-Pro Plus 6.0 image analysis software was used to analyze the results. Images are representative of each group. **P<0.01 vs. NG; ^∆^P<0.05 and ^∆∆^P<0.01 vs. HG. NG, normal group; MG, mannitol group; HG, high glucose/hypoxia group; AG, allicin group; GG, PKC inhibitor (GF109203x) group.

ROS production in the endothelial cells of the five groups was assessed by DHE staining. DHE is a fluorescent dye that specifically reacts with intracellular ROS and is converted to the red fluorescent compound ethidium, which then binds irreversibly to double-stranded DNA and appears as nuclear staining. As shown in Fig. 4, the intensity of DHE fluorescence in the endothelial cells in the HG was significantly enhanced compared with that in the NG; allicin significantly downregulated the levels of ROS (P<0.01).

#### Protein levels of 8-OHdG, NF-κB, Nox4 and HIF-1α and activity of PKC

**Figure 5.**
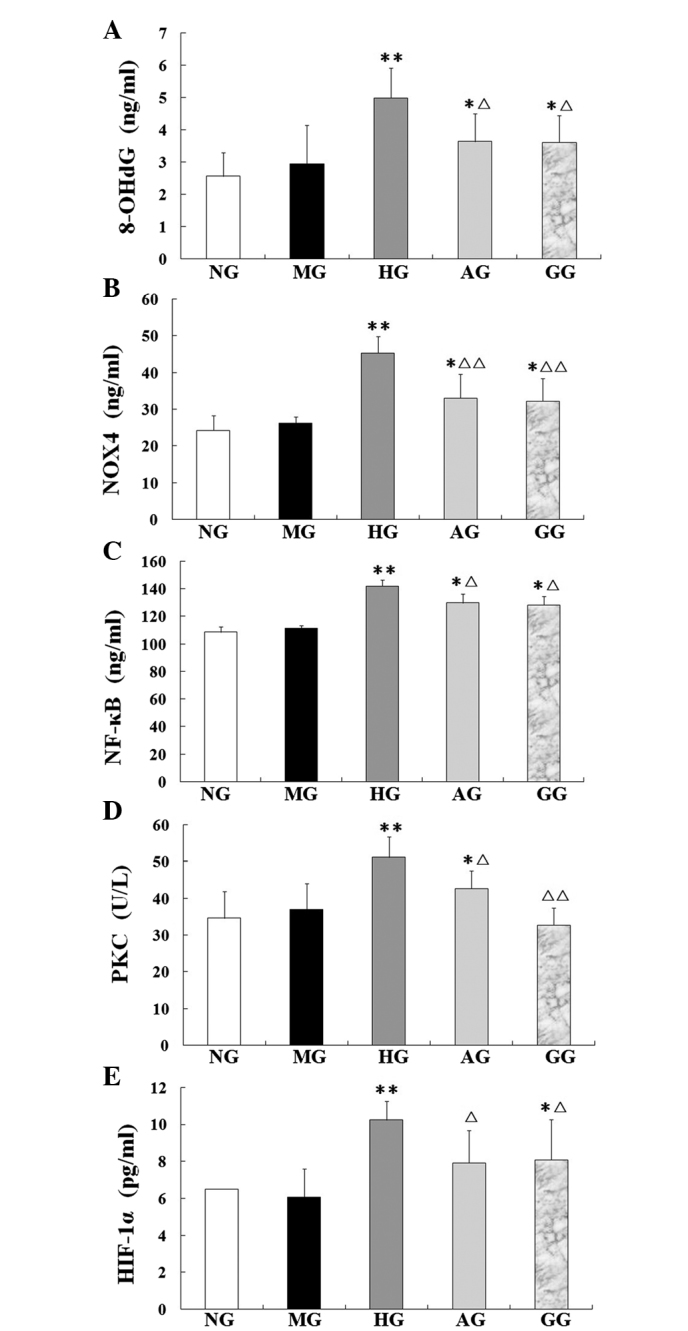
Levels of (A) 8-OHdG, (B) Nox4, (C) NF-κB, (D) PKC and (E) HIF-1α in aortic endothelial cells treated with different interventions. Data are presented as the mean ± standard deviation (n=10 per group). *P<0.05 and **P<0.01 vs. NG; ^∆^P<0.05 and ^∆∆^P<0.01 vs. HG. NG, normal group; MG, mannitol group; HG, high glucose/hypoxia group; AG, allicin group; GG, PKC inhibitor (GF109203x) group; 8-OHdG, 8-hydroxydeoxyguanosine; Nox4, NADPH oxidase 4; NF-κB, nuclear factor-κB; PKC, protein kinase C; HIF-1α, hypoxia-inducible factor-1α.

In the HG, the protein levels of 8-OHdG, NF-κB, Nox4 and HIF-1α and the activity of PKC were significantly increased compared with those of the NG (all P<0.01). In the AG, however, the five parameters were notably restored, showing a significant decrease compared with the levels in the HG (P<0.05 or P<0.01) (Fig. 5).

#### mRNA expression of NF-κB, Nox4 and HIF-1α

**Figure 6.**
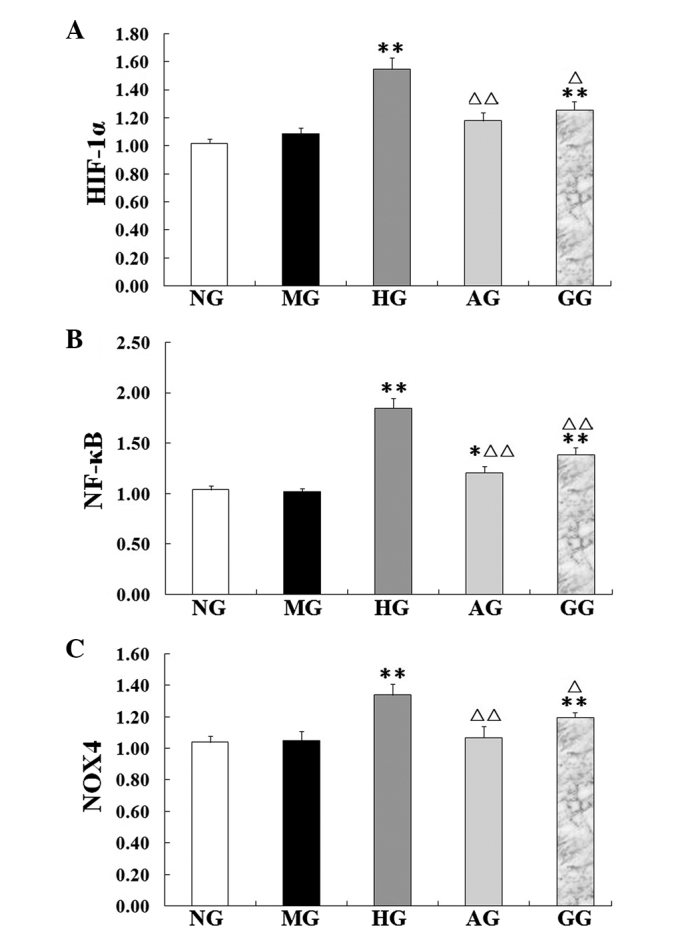
mRNA expression of (A) HIF-1α, (B) NF-κB and (C) Nox4 in aortic endothelial cells examined by a reverse transcription-quantitative polymerase chain reaction. Data are presented as the mean ± standard deviation (n=3 per group). *P<0.05 and **P<0.01 vs. NG; ^∆^P<0.05 and ^∆∆^P<0.01 vs. HG. NG, normal group; MG, mannitol group; HG, high glucose/hypoxia group; AG, allicin group; GG, PKC inhibitor (GF109203x) group; HIF-1α, hypoxia-inducible factor-1α; Nox4, NADPH oxidase 4; NF-κB, nuclear factor-κB.

In the HG, the NF-κB, Nox4 and HIF-1α mRNA expression was found to be significantly increased compared with the expression in the NG (P<0.01), which suggested that high glucose/hypoxia could induce the expression of these mRNAs (Fig. 6). Compared with the HG, however, the mRNA expression of NF-κB, Nox4 and HIF-1α in the AG was significantly decreased (P<0.05 or P<0.01).

## Discussion

In diabetes mellitus, the risk of atherosclerosis is enhanced, which results in an increase in the incidence of both cardiovascular and cerebrovascular diseases, such as myocardial infarction and cerebral ischemic attacks. Cardiovascular disease is the major cause of morbidity and mortality in diabetic patients (16). The exact mechanisms responsible for this accelerated development of atherosclerosis in diabetes have remained elusive, but oxidative stress appears to play a major role. Several studies have reported that the overproduction of ROS by the mitochondrial electron transport chain is responsible for hyperglycemia-induced oxidative damage and the pathogenesis of diabetic complications (17–21). Hyperglycemia promotes glucose oxidation and protein glycation, impairs DNA repair, with resultant DNA cleavage, and generates ROS, thereby leading to increased oxidative stress (22). Oxidative stress is considered to be the final common pathway through which the hyperglycemia-related pathways (PKC and polyol) can trigger the chronic complications of diabetes. Cumulative oxidant-mediated damage and cellular dysfunction are a result of an imbalance between ROS generation and antioxidant capacity. Furthermore, studies have found that there is a hypoxic microenvironment in diabetes, which is closely associated with the oxidative stress induced by hyperglycemia and plays an important role in diabetic complications (Amandine, Chavez). The development of therapeutic strategies aimed at the removal of free radicals or the prevention of their formation is therefore necessary.

To determine the injury elicited in aortic endothelial cells by high glucose/hypoxia and investigate whether allicin would effectively ameliorate the damage, the expression of oxidative stress-related markers was examined in endothelial cells cultured in high glucose (25 mmol/l for 24 h) and hypoxia [X/XO (1 mmol/l/20 U/l) for 40 min], simulating a diabetic microenvironment *in vivo*. The possible associated mechanisms were also explored. Since GF109203x is a highly specific PKC inhibitor, it was selected for use as a control. The study showed that the cells in the HG became shrunken and intercellular connections were lessened; furthermore, some of the cells became exfoliated and a few of the cells were observed to be floating. The MG was established to counteract the possible cellular injury by hyperosmosis, and it was found that the cell morphology of the MG was not obviously changed compared with that of the NG. Compared with the HG, the cells in the AG had more complete cell bodies with visible processes, which indicated that allicin played a protective role in the injured cells. Consistent with the MTT results, it was observed that allicin significantly inhibited cell death. Furthermore, a relatively abundant generation of ROS was measured in the cells of the HG by the DHE fluorescence probe. As shown in Fig. 4, allicin significantly downregulated the levels of ROS, showing its effective attenuation of the oxidative stress. The increase in ROS production observed in the endothelial cells of the HG was associated with an increase in the generation of Nox4 and 8-OHdG. NADPH oxidases of the Nox family are major sources of ROS, and the level of 8-OHdG is considered to be a marker of oxidative DNA damage (23). Our preliminary results confirmed the enhancement of oxidative damage to DNA in the HG; however, the damage was markedly attenuated when the cells were treated with allicin. These data suggested that allicin had signiﬁcantly protective antioxidative effects against high glucose/hypoxia-induced injury in aortic endothelial cells.

To the best of our knowledge, the persistent upregulation of PKC is recognized as an initial event leading to insulin resistance, cardiac disease and nephropathy in diabetes. Numerous studies (24–26) have previously reported that multiple PKC isoforms are activated in the vascular tissue of diabetic animal models, and the overactivation of the PKC pathway is a key mediator of diabetic vascular complications. The understanding of how hyperglycemia-induced oxidative stress ultimately leads to tissue damage has been advanced considerably (27), and strategies to reduce oxidative stress may exert favorable effects on the progression of diabetes; however, effective therapy to prevent or delay the development of this damage remains limited (28). The present results revealed that the level of PKC protein significantly increased in the murine aortic endothelial cells under high-glucose/hypoxia conditions, and the increase was mitigated by allicin, which demonstrated that the protective effects of allicin against the injury induced by high glucose/hypoxia involved the inhibition of the PKC pathway. PKC has been noted to contribute to the activation of NADPH oxidases in multiple cells (29). The Nox system is considered to be a key contributor to the generation of ROS in numerous cell types and tissues (30). Increased generation of ROS and impaired antioxidant defenses contribute to oxidative stress in diabetes (21). Previous studies have shown increased levels of NADPH oxidase subunits in the vasculature and kidney tissue of diabetic rodents (31,32). Nox4 was initially identified as a kidney NADPH oxidase, but it has since been shown that Nox4 is also abundant in vascular cells (33). There is additionally evidence that Nox4 contributes to oxidative stress in cardiovascular diseases (34,35). The present study showed a significant increase in Nox4 levels in the HG compared with those in the NG. Allicin significantly reduced the Nox4 mRNA and protein expression. These results suggest that high glucose/hypoxia increased the Nox4 levels, which caused an imbalance between the production of free radicals and the antioxidant defense system. Collectively, these data indicate that high-glucose conditions induce increased oxidative stress by activating the PKC pathway, which in turn mediates the increase in NADPH oxidase activity and Nox4 upregulation. Allicin can inhibit the PKC pathway, decrease Nox4-derived ROS and the endothelial cell injury induced by oxidative stress. The effects of allicin in the present study were similar to those of the PKC inhibitor GF109203x.

NF-κB is a pleiotropic, oxidant-sensitive transcription factor that is required for the transcription of the majority of proinflammatory molecules, including adhesion molecules, enzymes, cytokines and chemokines, which mediate the recruitment and retention of monocytes in the subendothelial space; a key early step in the atherosclerotic process. It has also been indicated that the activation of PKC is involved in the hyperglycemia-induced sustained activation of the transcription factor NF-κB (36). In the present study, as shown in Figs. 4 and 5, a significant increase was triggered by high glucose/hypoxia not only in the NF-κB mRNA level, but also in the NF-κB protein expression. Based on these findings, it is indicated that high-glucose conditions induced the activation of the transcription factor NF-κB through the upregulation of PKC. The upregulation of the PKC pathway leads to the concurrent upregulation of NADPH oxidase and increase in ROS production, which can damage endothelial cells and induce the expression of NF-κB, mediating the recruitment and retention of numerous inflammatory factors that further aggravate oxidative stress. Allicin can downregulate both the PKC activation and NF-κB expression. The inhibition of the PKC pathway should be considered as a target for the treatment of diabetes and its associated complications, and the present study showed that allicin inhibited the PKC pathway with a similar efficacy to that of the PKC inhibitor GF109203x.

Hypoxia has a prominent effect on all diabetic complications (37). HIF-1α is highly labile under normal oxygen conditions; however, under hypoxia it is strongly stabilized by ROS, preventing its hydroxylation and proteasomal degradation (38,39). In the present study, X/XO was used to establish the oxygen radical production system, simulating hypoxic conditions. HIF-1-dependent gene regulation leads to an adaptation to the hypoxic state, allowing the cell to survive. Anoxia, however, can induce the overexpression of HIF-1α, which leads to apoptosis-related gene expression, resulting in cell injury. In the present study, it was found that, dependent on the extent of the high glucose and hypoxia, HIF-1α levels also significantly increased. These results showed that endothelial cells incubated under high-glucose/hypoxic conditions expressed significantly high levels of HIF-1α, which were a risk factor for vascular lesion. In addition, allicin downregulated the expression of HIF-1α.

A broad range of antioxidant properties have been attributed to allicin, including direct scavenging of free radicals, maintenance of glutathione and the endogenous antioxidant redox balance, as well as the enhanced expression of the antioxidant enzymes glutathione peroxidase, glutathione reductase, SOD and CAT (13). Furthermore, allicin can suppress the production of certain inflammatory cytokines that have been implicated in the pathogenesis of diabetic complications, including tumor necrosis factor-α and transforming growth factor-1 (40). Specifically, a decrease in oxidative stress has been associated with reduced diabetes-associated atherosclerotic plaque development. As oxidative stress is associated with diabetic complications, antioxidation may be a key factor in the treatment of these diseases. The results of the present study indicate that allicin exerted beneficial effects on the treatment of macroangiopathy and could inhibit endothelial cell apoptosis and promote cellular survival under high-glucose/hypoxic conditions. These data offer a plausible explanation for the effects of allicin *in vivo*, which appear to be mediated at least partially by the inhibition of the PKC pathway and the consequent decrease in ROS production. In conclusion, the results of the present study suggest that allicin exerts protective effects via the inhibition of the high-glucose/hypoxia-induced upregulation of the PKC pathway and the subsequent activation of NADPH oxidase, increased ROS production, endothelial cell injury, 8-OHdG release and NF-κB upregulation. Inhibition of the PKC pathway may be the common protective mechanism of allicin in diabetes.
